# Adverse Reactions to Drugs of Special Interest in a Pediatric Oncohematology Service

**DOI:** 10.3389/fphar.2021.670945

**Published:** 2021-05-05

**Authors:** Kristopher Amaro-Hosey, Immaculada Danés, Lourdes Vendrell, Laura Alonso, Berta Renedo, Luis Gros, Xavier Vidal, Gloria Cereza, Antònia Agustí

**Affiliations:** ^1^ Clinical Pharmacology Service, Vall d’Hebron University Hospital, Barcelona, Spain; ^2^ Department of Pharmacology, Therapeutics and Toxicology, Universitat Autònoma de Barcelona, Barcelona, Spain; ^3^ Vall d’Hebron Research Institute, Barcelona, Spain; ^4^ Department of Pediatric Hematology and Oncology, Vall d’Hebron University Hospital, Barcelona, Spain; ^5^ Pharmacy Service, Vall d’Hebron University Hospital, Barcelona, Spain; ^6^ Catalan Institute of Pharmacology Foundation, Vall Hebron University Hospital, Barcelona, Spain

**Keywords:** pharmacovigilance, adverse drug reactions, pediatrics, hematology, neoplasms, pegaspargase, thioguanine, incidence

## Abstract

**Introduction:** Drugs used in oncological diseases are frequently related to adverse drug reactions (ADR). Few studies have analyzed the toxicity of cancer treatments in children in real practice.

**Methods:** An observational, longitudinal and prospective study has been carried out in an Oncohematology Service of a tertiary hospital. During 2017, patients exposed to one or more drugs of a previously agreed list were identified and followed-up for at least 6 months each. Characteristics of ADR, incidence, causality and possible preventability, have been evaluated.

**Results:** 72 patients have been treated with at least one study drug, and 159 ADR episodes involving at least one of these drugs have been identified, with a total of 293 ADR. Most episodes required hospital admission (35.2%) or happened during the hospital stay (33%), and 91.2% were severe. Blood disorders were the most frequent ADR (96; 32.8%), related to thioguanine (42) and pegaspargase (39) mainly, followed by infections (86; 29.4%) related to thioguanine (32), pegaspargase (27), *Erwinia* asparaginase (14) and rituximab (13). Two ADR were unknown. Most ADR were dose-dependent or expectable (>90%). The global incidence of ADR was 3.1/100 days at risk (95% CI 2.7–3.5), with 3.5 ADR/100 days at risk with pegaspargase (95% CI 2.9–4.2), 1.2/100 days at risk with rituximab (95% CI 0.8–1.8) and 11.6/100 days at risk with thioguanine (95% CI 9.4–14.2). Controversial additional measures of prevention, other than those already used, were identified.

**Conclusion:** ADR are frequent in pediatric oncohematological patients, mainly blood disorders and infectious diseases. Findings regarding incidence and preventability may be useful to compare data between different centers and to evaluate new possibilities for action or prevention.

## Introduction

Adverse drug reactions (ADR) are a major cause of morbidity and mortality in adults and children. Between 2 and 5% of hospital admissions in children are due to ADR (Impicciatore et al., 2001), and its incidence among hospitalized pediatric population has been described as 0.6–17.7% (Gallagher et al., 2011; Stausberg and Hasford., 2011; Gallagher et al., 2012; Posthumus et al., 2012; Thiesen et al., 2013). The highest percentage was documented in a study carried out in a United Kingdom pediatric hospital, in which the oncological treatment was identified as a risk factor for ADR (HR 1.90; 95% CI 1.40–2.60) (Thiesen et al., 2013). In spite of that, there are few studies in which the impact of oncological treatments in children has been quantified or analyzed, and their methodology has varied. A retrospective study including a random sample of adult and children hospitalized at a university oncology centre showed that 22% of ADR happened in patients younger than 20 (Vaseghi et al., 2016). Cisplatin (44%), doxorubicin (24%) and 5-fluouracil (20%) were the chemotherapeutic drugs most commonly involved and the principal symptoms were nausea, vomiting, neutropenia and constipation, both in adults and in pediatric population. With these results the authors could identify prevention strategies to reduce their incidence. Other studies have been focused on validating indirect tools or systems for detecting possible preventable unwanted effects in oncohematology services by identifying drug use (protamine, vitamin K, naloxone, flumazenil or hyaluronidase) commonly prescribed when ADR with certain medications occur (Call et al., 2014; Hébert et al., 2015). Other factors that may affect toxicity are the use of recently marketed drugs with incomplete knowledge of the toxicity profile and off-label drug use (Bellis et al., 2013; Saiyed et al., 2015).

At the end of 2013, the European Union initiated a new procedure to identify drugs undergoing particularly rigorous monitoring by health authorities (Agencia Española de Medicamentos y Productos Sanitarios, 2013; European Medicines Agency, 2013). These drugs are subject to “additional monitoring”, and a black inverted triangle is displayed in their package leaflet and in the product summary, together with a short sentence explaining the triangle meaning. This is generally because there is less information available on it than on other medicines, for example because it is new to the market or there is limited data on its long-term use. This includes, for example, new active ingredients, including biological medicines, authorized for the first time in the EU after January 1, 2011, medicines to which a conditional marketing authorization has been granted, have been approved in exceptional circumstances, or have been authorized with specific obligations on the recording or suspected ADR, sometimes based on advice from the Agency’s Pharmacovigilance Risk Assessment Committee (PRAC).

For this study the knowledge of the incidence and characteristics of ADR related to medications of special interest in safety matters in a third level hospital was prioritized. The main objective of the study was to evaluate the incidence and characteristics of suspected ADR to medications of special interest, with the final purpose of knowing better its safety profile in pediatric population in clinical practice. As secondary objectives, we wanted to identify possible preventive measures, to analyze the proportion of patients with ADR in whom the use of the drugs was off-label and to improve the identification of suspected ADR in the Pediatric Oncohematology service.

## Article Types

Original Research.

## Methods

An observational, longitudinal and prospective study was carried out in the Pediatric Oncohematology Service of Vall d’Hebron University Hospital. After reviewing drug consumption during 2016 in the Pediatric Oncohematology Service, a list of drugs with an incomplete knowledge about their safety profile in the general population or specifically in children was identified and agreed between all researchers, and selected to be monitored. Drugs selected were those used during the previous year with one or more of the following characteristics: marketed in exceptional circumstances, newly authorized with conditional approval, subject to special follow up by the European Medicines Agency (EMA), not marketed in Spain, and drugs frequently used off-label or with little experience in children. Based on these criteria, the following drugs were selected: clofarabine, nelarabine, thioguanine, crizotinib, pegaspargase, asparaginase, defibrotide, infliximab, rituximab, bevacizumab, imatinib, dasatinib, nilotinib, eltrombopag and romiplostim. It is important to note that some drugs such as thioguanine, defibrotide and asparaginase are not marketed in Spain, and neither was pegaspargase when the study began.

During 2017, all patients exposed to at least one drug of the list were identified at the Pharmacy Service based on the prescription requests received daily, and were followed for a minimum of 6 months in order to detect possible ADR. Patients with at least one ADR suspicion were identified using different sources (notification by clinicians attending the patients and/or patients monitoring throughout the clinical records). ADR were grouped as an episode when more than one ADR coincided in time in the same patient, and the suspected drugs (even in polytherapy) and involved mechanisms were also considered coincident when assessing the causal relationship according to the pharmacovigilance methods used (detailed in the next paragraph). This allows to have detailed information according to ADR and also about events or clinical situations with ADR. For example, if a patient had thrombocytopenia and hemorrhage, or febrile neutropenia and a certain infection, two ADR were considered but only one ADR episode in each case. The number and characteristics of ADR episodes were registered. Cytopenias were only recorded if they caused some additional medical act such as tests or treatments such as antibiotics, transfusions or a delay in chemotherapy administration. Demographic data, clinical diagnosis, preventive measures used and other variables necessary to evaluate ADR causality were also obtained from the review of the medical records. Information on prior knowledge of ADR suspicions was obtained from drug datasheets and the review of the published bibliography. Use was considered off-label if indication, population or dosage were different from the medication data sheet approved by the regulatory authority.

ADR were classified according to the Medical Dictionary for Regulatory Activities MedDRA® (Medical Dictionary for Regulatory Activities, 2020). The plausibility of the association between the ADR and drugs has been analyzed and discussed, according to the Spanish Pharmacovigilance System algorithm, which takes into account the temporal sequence, prior knowledge of the adverse reaction to the drug, drug withdrawal effect, drug re-exposure effect (if available), and possible alternative causes of symptoms (Aguirre and García, 2016). It has also been used to classify previous knowledge of the ADR in well-known ADR, known from anecdotal reports and unknown. The seriousness of the ADR was classified in accordance with to the European Union’s criteria: ADR were considered serious (when they result in death, were life-threatening, required hospitalization or prolonged an existing hospitalization, resulted in persisting disability or were important medical events) or non-serious (the remaining cases) (European Medicines Agency, 2004).

Incidence of ADR was calculated taking into account the number of days of exposure to each drug. Time at risk with each drug was considered taking into account the interval for drug administration (related to pharmacokinetic and pharmacodynamic parameters) of drug as the main guide. Exposure was considered continued during all treatment as long as the normal interval of drug administration was maintained, and when a longer interruption happened or the treatment ended, the same number of days after the last dose were considered as a time at risk. Normal interval for administration is 14 days for pegaspargase, 2 days for *Erwinia* asparaginase, seven for romiplostim, twenty-one for nelarabine (with a short half-live but high intracellular accumulation of ara-GTP), 8 weeks (60 days) for infliximab and 6 months for rituximab (the interval of administration in some indications, when the CD20 cells recovery starts) (Yun et al., 2015; Aaltonen et al., 2015; European Medicines Agency, 2021). For the rest of drugs, administered daily, the treatment period was considered. When one patient was exposed to more than one study drug and time at risk coincided at some point, the same time at risk was considered for each drug to calculate incidence data. An electronic database was set up and analyzed with the statistical package SAS® 9.4 (SAS Institute Inc., Cary, NC, United States). Continuous and count variables were summarized using medians with 25th and 75th percentiles, and categorical factors were reported using frequencies and percentages. Incidence rates were calculated by dividing the number of ADR by the corresponding time at risk, and expressed for 100 days at risk. 95% confidence intervals were estimated from Poisson distribution.

The study was conducted according to international ethical recommendations and was approved by the local Research Ethics Committee following the national directives related to post-authorization studies.

## Results

A total of 72 patients treated with at least one study drug were identified. Demographic characteristics of these patients and medical conditions for which study drugs were used are shown in Table 1. Patients were followed for a median of 388.5 days (IQR 252–483.5). Table 2 shows the distribution of drugs and data of exposure. Forty-five patients (62.5%) were exposed to more than one study drug. There were no cases exposed to nilotinib, crizotinib, clofarabine or bevacizumab. The drugs with a longer total time at risk were pegaspargase (3,539 days), rituximab (2,051), thioguanine (794), eltrombopag (768) and imatinib (597). Study drugs were used to treat 10 different diseases; the most frequent were acute lymphoblastic leukemia (43), hepatic veno-occlusive disease (12) and Epstein-Barr virus infection (7). Thirty-three percent of patients with leukemia had a high-risk disease.

**TABLE 1:** Baseline characteristics of the cohort of patients exposed to study drugs.

	Patients (*n* = 72)
Age (median [IQR]) years	6 (3.1–10.2)
<2 years, *n* (%)	9 (12.5%)
2–12 years, *n* (%)	50 (69.4%)
>12 years, *n* (%)	13 (18.1)
Gender	
Female, *n* (%)	29 (40.3%)
Male, *n* (%)	43 (59.7%)
Exposition to study drugs^a^	
1 drug, *n* (%)	27 (37.5)
2 drugs, *n* (%)	37 (51.4)
3 drugs, *n* (%)	8 (11.1)
Indication of study drugs (*n*)	
Acute lymphoblastic leukemia	43
Hepatic veno-occlusive disease	12
Epstein-Barr virus infection	7
Idiopathic thrombocytopenic purpura	5
Lymphoblastic lymphoma	3
Autoimmune hemolytic anemia	3
Chronic graft-versus-host disease	2
Chronic myeloid leukemia	1
Dermatofibrosarcoma protuberans	1
Aplastic anemia	1

a Fourteen out of 37 patients exposed to 2 study drugs (pegaspargase and thioguanine in eight patients) had some period of coincidence of risk time.

**TABLE 2:** Exposure to study drugs.

Drugs	Time at risk considered^a^	Number of exposed patients	Total number of days of exposure	Median number of days of exposure (IQR)
Pegaspargase	During treatment at normal interval +14 days	38	3,539	63 (42–240)
Thioguanine	During treatment at normal interval	37	794	15 (14–25)
Defibrotide	During treatment at normal interval	12	320	25.5 (16.5–31.5)
*Erwinia* asparaginase	During treatment at normal interval +2 days	11	384	24 (24–36)
Rituximab	During treatment at normal interval +180 days	10	2,051	194 (188–201)
Eltrombopag	During treatment at normal interval	5	768	113 (67–178)
Romiplostim	During treatment at normal interval +7 days	3	346	62 (56–228)
Nelarabine	During treatment at normal interval +21 days	2	102	51 (50–52)
*E. coli* asparaginase	During treatment at normal interval	2	16	8 (4–12)
Imatinib	During treatment at normal interval	2	597	298.5 (113–484)
Infliximab	During treatment at normal interval +60 days	2	168	84 (60–108)
Dasatinib	During treatment at normal interval	1	402	402 (402–402)

a Additional days were considered when treatment ended or an interruption longer than the interval of administration happened.

Fifty-two out of 72 patients (72.2%) presented some ADR in which a study drug was considered suspicious. Median age of these patients was 6.8 years (IQR 3.4–11.5) and 58% were male. The total number of ADR episodes was 159, with a median of two episodes per patient (IQR 0–3), and 3 (IQR 2–6) when analysis was performed only in patients with high-risk leukemia. Most episodes (90.5%) were identified by monitoring clinical records. Fifty-six episodes required hospital admission (35.2%), 33.3% happened during the hospital stay, and 31.5% were managed on an outpatient basis. The majority of episodes of ADR were serious (145; 91.2%): the principal reasons for severity were the requirement of hospitalization or prolongation of an existing hospitalization (60; 37.7% of episodes) and the consideration of the episode medically important (60; 37.7% of episodes). Eight episodes were life-threatening, but no deaths due to adverse effects were registered.

Two-hundred ninety-three ADR were described in the 159 episodes. Patients were receiving a median of seven drugs per episode (IQR 6–9) and were considered suspicious a median of 3 (IQR 2–4). At least one drug of the study list was involved in these reactions, being pegaspargase and thioguanine the most frequent (Table 3). Fifteen percent of suspicious study drugs had been given off-label, mainly rituximab for Epstein-Barr virus infection (on 12 different occasions) or for autoimmune hemolytic anemia (4). As shown in Table 4, the principal ADR with study drugs were blood disorders related to thioguanine, pegaspargase and *Erwinia* asparaginase, infections with thioguanine, pegaspargase, *Erwinia* asparaginase and rituximab, and gastrointestinal or metabolic disorders with pegaspargase (additional information is available as Supplementary Material). Specifically, the most reported ADR (accounting for almost 40% of ADR) were febrile neutropenia, pancytopenia, thrombocytopenia, fever, hypertriglyceridemia and agranulocytosis. Suspicious drugs considered possibly involved in these ADR are detailed in Table 5.

**TABLE 3:** Number of ADR with study drugs as suspects.

Drugs	Number of ADR
Pegaspargase	124 (39.2)
Thioguanine	92 (29.2)
*Erwinia* asparaginase	47 (14.9)
Rituximab	25 (7.9)
Defibrotide	9 (2.9)
*E. coli* asparaginase	6 (1.9)
Eltrombopag	4 (1.3)
Dasatinib	3 (1.0)
Nelarabine	2 (0.6)
Imatinib	2 (0.6)
Infliximab	1 (0.3)
Romiplostim	0

**TABLE 4:** Principal ADR according to the affected system and suspicious study drugs^a^.

ADR	*n* (%)	Suspicious study drugs	Other suspicious drugs in ≥5 ADR
Blood and lymphatic system disorders	96 (32.8)	Thioguanine (42), pegaspargase (39), *Erwinia* asparaginase (19), defibrotide (3), rituximab (3), dasatinib (2), *E. coli* asparaginase (2)	Cytarabine (53), cyclophosphamide (37), vincristine (38), dexamethasone (36), methotrexate (20), mercaptopurine (15), doxorubicin (16), daunorubicin (8), etoposide (8), methylprednisolone (6)
Infections and infestations	86 (29.4)	Thioguanine (32), pegaspargase (27), *Erwinia* asparaginase (14), rituximab (13), *E. coli* asparaginase (2), defibrotide (1), eltrombopag (1), infliximab (1)	Cytarabine (38), vincristine (29), cyclophosphamide (24), dexamethasone (23), methotrexate (18), mercaptopurine (13), daunorubicin (10), methylprednisolone (10), doxorubicin (9), mycophenolic acid (6)
Gastrointestinal disorders	23 (7.8)	Pegaspargase (11), thioguanine (6), *Erwinia* asparaginase (5), *E. coli* asparaginase (1)	Cytarabine (11), dexamethasone (11), vincristine (11), methotrexate (10), cyclophosphamide (7), mercaptopurine (6)
Metabolism and nutrition disorders	22 (7.5)	Pegaspargase (21), *Erwinia* asparaginase (1)	Dexamethasone (11)
General disorders and administration site conditions	16 (5.5)	Thioguanine (7), pegaspargase (6), rituximab (2), *Erwinia* asparaginase (1), *E. coli* asparaginase (1), imatinib (1)	Cyclophosphamide (8), cytarabine (8), vincristine (6), dexamethasone (5)
Skin and subcutaneous tissue disorders	13 (4.4)	Pegaspargase (9), *Erwinia* asparaginase (2), defibrotide (1), thioguanine (1)	
Respiratory, thoracic and mediastinal disorders	10 (3.4)	Rituximab (4), thioguanine (3), pegaspargase (2), *Erwinia* asparaginase (1), nelarabine (1)	
Other	27 (9.2)		

a More than one drug could be involved in an ADR.

**TABLE 5:** ADR and suspicious drugs^a^.

ADR according to the affected system	*n* (%)	Suspicious study drugs	Other suspicious drugs in ≥5 ADR
Febrile neutropenia	27 (9.2)	Thioguanine (11), pegaspargase (9), *Erwinia* asparaginase (8), *E. coli* asparaginase (1)	Vincristine (16), cytarabine (15), dexamethasone (13), cyclophosphamide (8), daunorubicin (6), doxorubicin (6), methylprednisolone (5)
Pancytopenia	25 (8.6)	Thioguanine (13), pegaspargase (12), *Erwinia* asparaginase (4)	Cytarabine (19), cyclophosphamide (14), dexamethasone (13), vincristine (10), methotrexate (6), doxorubicin (5)
Thrombocytopenia	22 (7.5)	Pegaspargase (10), thioguanine (9), *Erwinia* asparaginase (4), defibrotide (2), dasatinib (1), rituximab (1)	Cytarabine (12), cyclophosphamide (8), methotrexate (6), mercaptopurine (5)
Fever	13 (4.4)	Thioguanine (7), pegaspargase (4), rituximab (2), *E. coli* asparaginase (1), *Erwinia* asparaginase (1)	Cyclophosphamide (8), cytarabine (8), vincristine (6), dexamethasone (5)
Hypertriglyceridemia	13 (4.4)	Pegaspargase (13)	
Agranulocytosis	12 (4.1)	Thioguanine (8), *Erwinia* asparaginase (3), pegaspargase (3)	Cyclophosphamide (7), cytarabine (6)
Respiratory tract infection	7 (2.4)	Pegaspargase (5), thioguanine (2), rituximab (1)	
Anemia	5 (1.7)	Pegaspargase (3), defibrotide (1), rituximab (1), thioguanine (1)	
Stomatitis	5 (1.7)	*Erwinia* asparaginase (2), pegaspargase (2), thioguanine (1)	
Respiratory tract viral infection	5 (1.7)	Pegaspargase (2), *Erwinia* asparaginase (1), rituximab (1), thioguanine (1)	
Rhinovirus infection	5 (1.7)	Thioguanine (3), pegaspargase (2), rituximab (1)	
Mucosal inflammation	5 (1.7)	Pegaspargase (2), thioguanine (2), *Erwinia* asparaginase (1)	
Other	149 (50.9)		

a More than one drug could be involved in an ADR.

Hypersensitivity/allergic reactions accounted for 9.5% of the episodes, while the rest of ADRs (90.5%) were considered dose-related or due to mechanisms other than hypersensitivity. For cytopenias and infections, the mechanism is well known; however, for other common complications such as hypertriglyceridemia or pancreatitis with pegaspargase, the mechanism is not fully established (genetic predisposition has been suggested) but hypersensitivity is highly unlikely (Zhang et al., 2020). The incidence of ADR was 3.1/100 days at risk (95% CI 2.7–3.5), and for episodes of ADR incidence was 1.7/100 days at risk (95% CI 1.4–2). The incidence of ADR with the drugs with a longer total time at risk in this study was: 3.5 ADR/100 days at risk with pegaspargase (95% CI 2.9–4.2), 1.2/100 days at risk with rituximab (95% CI 0.8–1.8) and 11.6/100 days at risk with thioguanine (95% CI 9.4–14.2). More specifically, the most frequent ADR with pegaspargase were blood disorders (1.1/100 days at risk; 95% CI 0.8–1.5) and infections (0.8/100 days at risk; 95% CI 0.5–1.1); with rituximab ADR were mainly infections (0.6/100 days at risk; 95% CI 0.4–1.1), and with thioguanine, blood disorders (5.3/100 days at risk; 95% CI 3.9–7.2) and infections (4/100 days at risk; 95% CI 2.9–5.7).

There were five episodes of ADR despite using specific prophylaxis to prevent them: three patients with herpes infection were receiving acyclovir and two patients with rituximab infusion-related disorders had received premedication with antihistamines and analgesics. No other prophylactic methods were applied in the rest of patients with ADR. The prevention of ADR according to individual susceptibility could have been carried out in 30 episodes of hematological adverse reactions to thioguanine by phenotyping of thiopurine methyltransferase. In eight episodes of ADR consisting of neutropenia/agranulocytosis in patients with high risk leukemia during high-risk blocks, prophylaxis with granulocyte colony-stimulating factor could have been considered.

ADR with drugs of the list were previously unknown or poorly known: papular acne related to *Erwinia* asparaginase and pneumatosis intestinalis related to pegaspargase. Additionally, during the follow-up of the global cohort of 72 patients exposed to some study drug, 179 episodes involving drugs different from study drugs were detected, with a median number of two episodes per patient (IQR 1–4). One-hundred forty-three (80%) were considered serious, mainly because hospitalization was required or were considered medically important (110; 61.5%). Two-hundred and ninety-five ADR were described in these episodes, all of them previously known. The principal ADR according to the affected system were infections and infestations (80; 27.1%), blood and lymphatic system disorders (73; 24.7%), gastrointestinal disorders (34; 11.5%), general disorders and administration site conditions (18; 6.1%), hepatobiliary disorders (17; 5.8%), and skin and subcutaneous tissue disorders (14; 4.7%). The most reported ADR were pancytopenia (56), anemia (46), stomatitis (38), thrombocytopenia (36), and hepatitis (35). Median number of suspicious drugs per episode was 2 (IQR 1–3). The drugs more commonly involved as suspicious were mercaptopurine (in 115 ADR), cytarabine (85), methotrexate (76), cyclophosphamide (51), dexamethasone (49), and cyclosporine (41). Additional information on the most frequent ADR and suspicious drugs is provided as Supplementary Material.

## Discussion

To our knowledge, this is the first prospective study in pediatric oncohematological population assessing the incidence and characteristics of ADR in real practice to specific drugs of interest in pharmacovigilance. This intensive 18 months follow-up study has shown that three out of four patients treated had at least one ADR to a study drug considered suspicious, with a mean number of two episodes per patient. Pegaspargase and thioguanine have been the drugs most frequently involved (68%), and blood disorders and infections were the most frequently reported ADR (78%).

Drugs used in cancer diseases are described as a risk factor of ADR occurrence, but only very few pharmacovigilance studies have assessed ADR in oncohematological pediatric patients. Methodology used in these studies is very heterogeneous and therefore, the results are also highly variable. Barret et al. described a 14.4–23.5% annual rate incidence of adverse events in a 7 years retrospective study by using databases (Barrett et al., 2013). Queuille et al., in a prospective 50 days study in inpatients, described at least one ADR in 65% of inpatients (Queuille et al., 2001). In a retrospective study carried out in pediatric cancer patients admitted at two hospitals in Ethiopia during a 2 years period, ADR were described in 41.5% of patients, with a higher risk in patients taking four or more chemotherapy agents and regimens based on etoposide, mercaptopurine, and doxorubicin (Workalemahu et al., 2020). In our study, a 72.2% (52 out of 72) ADR incidence was described, however our population was limited to a list of specific drugs and follow-up time was different. Also, we have been able to assess incidence rates during time at risk of significant blood disorders or infections in clinical practice during treatment with specific drugs such as pegaspargase, rituximab, and thioguanine even though, like in a clinical trial context, these drugs were not given alone and other drugs can have contributed to these incidence data.

Fifteen percent of the study drug treatments were used off-label, a smaller proportion than expected. The drugs administered off-label were different from the drugs used according to the labeling. For both reasons, the comparison of the incidence of ADR between the use of off-label and on-label drugs was considered inappropriate. A similar trend was documented in the study of Mascolo et al., which aimed to assess off-label use of Individual Case Safety Reports concerning antineoplastic drugs in children and found that only 18 (7.6%) out of 236 were classified as off-label cases (Mascolo et al., 2020). These authors considered the low number of cases were due to underreporting.

Rituximab was the most frequently drug used off-label. In children, rituximab has been used in a wide range of autoimmune diseases that require lymphocyte B depletion, and observational studies have been published in patients with immune thrombocytopenia, hemolytic anemia, multiple sclerosis or nephrotic syndrome (Zecca et al., 2003; O’Connor et al., 2013; Maratea et al., 2016; Makis et al., 2018). The main indications for rituximab in our cohort were the treatment of Epstein-Barr virus infection and hemolytic anemia. Weekly administrations of 1–3 doses were used and the principal ADR described during the follow-up were infections. In studies published with rituximab use to prevent Epstein-Barr virus related post-transplant lymphoproliferative disorders in allogeneic hematopoietic stem cell transplantation, little information is provided on specific side-effects of rituximab (Lindsay et al., 2020; Kiskaddon et al., 2021). In one study with 78 patients with Epstein-Barr virus reactivation treated with rituximab, a higher 36-month cumulative incidence of bacterial, viral, and fungal infections was described compared to a control group of patients not treated with rituximab, and a slower reconstitution of B cells was observed (Petropoulou et al., 2012). In other series of patients with autoimmune hemolytic anemia, infective complications were unusual, probably because patients were less exposed to concomitant cytotoxic drugs than those with hematological malignancies in whom Epstein-Barr virus was detected, and possibly because immunoglobulin replacement therapy was administered. In a cohort of 61 children with autoimmune hemolytic anemia treated with weekly infusions of rituximab for four weeks, two cases of allergic reactions were described, one patient died due to agranulocytosis and sepsis probably related with rituximab treatment, and 16 cases of hypogammaglobulinemia were identified, and partially treated with immunoglobulin replacement in 13 of them for at least 5 months (Ducassou et al., 2017). In another prospective study in 15 children with autoimmune hemolytic anemia, three moderate side effects during rituximab infusion and one case of varicella zoster infection 2 months after treatment were described (Zecca et al., 2003). In this study, although benefits and risks of this practice have not been proven, all children received intravenous immunoglobulin for 6 months to prevent hypogammaglobulinemia after completing treatment.

Nine out of ten ADR episodes were considered severe; no other study has evaluated the severity of ADRs in pediatric oncohematological patients taking into account current criteria of severity (European Medicines Agency, 2004). The proportion of episodes that required hospital admission, occurred during the hospital stay, or on an outpatient basis was similar. In studies with a different approach including only children with oncohematological diseases admitted to the hospital, the proportion of ADR that caused admission during a specific period was 41% compared to 59% that occurred during hospitalization (Collins et al., 1974). If in our study we exclude patients managed on an outpatient basis, these percentages are quite similar: 51 and 49%, respectively. Only 9.5% of episodes of ADR were considered due to hypersensitivity in our study compared to nearly 20% of hospital-acquired ADR in the study by Collins et al., with different drugs and methods.

The ADR profile identified indicates a clear predominance of hematologic and infectious reactions; also, some characteristic ADR such as hypertriglyceridemia due to pegaspargase has been registered frequently. Gastrointestinal disorders have been less frequent with mucositis being the most common one (oral or anal mucositis accounted for 61% of gastrointestinal disorders). Only two cases of vomiting were identified (and no cases of nausea). Neutrophil count decreases and alanine aminotransferase increases represented the most frequently identified ADR in a study focused on the identification of ADR with drug combinations (Barrett et al., 2013). In an old study, Collins et al. reported vomiting as the principal ADR (Collins et al., 1974). Drug characteristics can be in part responsible for this difference, but also the fact that chemotherapy-related nausea and vomiting prevention has improved substantially. On the other hand, prevention of hematological toxicity and infections are now goals not fully addressed. Some thioguanine-related hematological ADR could possibly had been prevented by implementing thiopurine methyltransferase testing (TMPT) in patients with leukemia (Lennard, 2014; Lennard et al., 2015). This test for now is not performed in our center and when the results sent to other centers are available the dose has already been adjusted. Regarding bacterial infections, different antibiotic prophylaxis schemes (including ciprofloxacin, levofloxacin or amoxicillin-clavulanate) have shown in clinical trials a reduction of episodes of fever and infections compared to placebo, but a significant reduction of mortality has not been achieved (Castagnola et al., 2003; Laoprasopwattana et al., 2013; Alexander et al., 2018). The expected risk of an increase of resistant strains prevents their recommendation as a routine. For this reason, antimicrobial prophylaxis (other than trimetroprim/sulphamethoxazole to prevent *Pneumocystis jirovecii* infection, acyclovir for herpes zoster prevention and antifungal in high-risk patients) is not used in our center either. Granulocyte colony-stimulating factor is only used prophylactically in high-risk blocks (SEHOP/PETHEMA, 2013).

One limitation of our study is the selection of patients treated with specific drugs. This has allowed a more intensive follow-up of treated patients and the possibility to assess incidence data of patients treated with these drugs, but prevents a fair comparison with other studies. In order to improve the drug safety analysis in children with oncohematological diseases, guidelines would be a good tool not only to assess ADR but also to compare incidence and prevalence of ADR between centers to identify areas for improvement. During and after the study, the identification of adverse reactions in general in the pediatric oncohematology service has increased compared to previous years. Although the effect is expected to be temporary, we believe that more awareness has been raised about ADR and the desirability of looking for the most appropriate way to make their identification useful. Multidisciplinary management and constant communication between teams should be encouraged in order to provide high quality health assistance. On the other hand, the study was not designed to assess the effect of measures of prevention but can be useful to discuss the convenience to review protocols for most risky patients and the possibility to include new techniques such TMPT testing in our hospital.

In conclusion, the main ADRs related to the study drugs in our study are blood disorders and infections related to treatments that include pegaspargase and thioguanine. The incidence data described can be useful to compare toxicity between different centers and to assess new possibilities for action or prevention, in order to reduce these risks and improve quality of life, always maintaining efficacy.

## Data Availability

The raw data supporting the conclusions of this article will be made available by the authors, without undue reservation.
