# Supplementary material for: Adverse Reactions to Drugs of Special Interest in a Pediatric Oncohematology Service
Source: Front Pharmacol. 2021 May 5;12:670945. doi: 10.3389/fphar.2021.670945 (PMC8131830; doi:10.3389/fphar.2021.670945)
Supplement: Supplementary file 1 [file DataSheet1.docx]

Supplementary Material

**Table 4 (completed). ADR according to the affected system and suspicious drugs***

| **ADR according to the affected system** | **n (%)** | **Suspicious study drugs** | **Other suspicious drugs** |
| --- | --- | --- | --- |
| Blood and lymphatic system disorders | 96 (32.8) | Thioguanine (42), pegaspargase (39), *Erwinia* asparaginase (19), defibrotide (3), rituximab (3), dasatinib (2), *E. coli* asparaginase (2) | Cytarabine (53), cyclophosphamide (37), vincristine (38), dexamethasone (36), methotrexate (20), mercaptopurine (15), doxorubicin (16), daunorubicin (8), etoposide (8), methylprednisolone (6), cyclosporine (4), mycophenolic acid (2), trimethoprim/sulfamethoxazole (2), busulfan (1), daunoblastin (1), fludarabine (1), hydrocortisone (1), mitoxantrone (1), piperacillin/tazobactam (1), prednisone (1), thiotepa (1) |
| Infections and infestations | 86 (29.4) | Thioguanine (32), pegaspargase (27), *Erwinia* asparaginase (14), rituximab (13), *E. coli* asparaginase (2), defibrotide (1), eltrombopag (1), infliximab (1) | Cytarabine (38), vincristine (29), cyclophosphamide (24), dexamethasone (23), methotrexate (18), mercaptopurine (13), daunorubicin (10), methylprednisolone (10), doxorubicin (9), mycophenolic acid (6), cyclosporine (4), hydrocortisone (3), etoposide (2), piperacillin/tazobactam (2), prednisone (2), ifosfamide (1), ruxolitinib (1), vindesine (1) |
| Gastrointestinal disorders | 23 (7.8) | Pegaspargase (11), thioguanine (6), *Erwinia* asparaginase (5), *E. coli* asparaginase (1) | Cytarabine (11), dexamethasone (11), vincristine (11), methotrexate (10), cyclophosphamide (7), mercaptopurine (6), doxorubicin (4), daunorubicin (2), ifosfamide (1), vindesine (1) |
| Metabolism and nutrition disorders | 22 (7.5) | Pegaspargase (21), *Erwinia* asparaginase (1) | Dexamethasone (11), methotrexate (2), methylprednisolone (2), daunorubicin (1), mercaptopurine (1), vincristine (1) |
| General disorders and administration site conditions | 16 (5.5) | Thioguanine (7), pegaspargase (6), rituximab (2), *Erwinia* asparaginase (1), *E. coli* asparaginase (1), imatinib (1) | Cyclophosphamide (8), cytarabine (8), vincristine (6), dexamethasone (5), doxorubicin (4), methotrexate (2), mycophenolic acid (1) |
| Skin and subcutaneous tissue disorders | 13 (4.4) | Pegaspargase (9), *Erwinia* asparaginase (2),  defibrotide (1), thioguanine (1) | Cytarabine (2), dexamethasone (2), mercaptopurine (2), methotrexate (2), vincristine (2), cyclophosphamide (1), heparin (1), mitoxantrone (1) |
| Respiratory, thoracic and mediastinal disorders | 10 (3.4) | Rituximab (4), thioguanine (3), pegaspargase (2), *Erwinia* asparaginase (1), nelarabine (1) | Cyclophosphamide (4), cytarabine (2), etoposide (1), mercaptopurine (1), methotrexate (1), cyclosporine (1) |
| Hepatobiliary disorders | 8 (2.7) | Pegaspargase (3), eltrombopag (2), *Erwinia* asparaginase (2), thioguanine (1) | Fluconazole (3), methotrexate (3), vincristine (2), amphotericin B (1), cyclophosphamide (1), cytarabine (1), daunorubicin (1), dexamethasone (1), doxorubicin (1), mercaptopurine (1), mesna (1), mycophenolic acid (1), posaconazole (1) |
| Nervous system disorders | 5 (1.7) | Pegaspargase (3), nelarabine (1), dasatinib (1) | Daunorubicin (3), vincristine (3), cyclophosphamide (1), etoposide (1), methylprednisolone (1) |
| Immune system disorders | 3 (1.0) | Rituximab (2), pegaspargase (1) | Mycophenolic acid (1) |
| Renal and urinary disorders | 3 (1.0) | Defibrotide (2), *Erwinia* asparaginase (1) | Cyclosporine (2), cytarabine (1), dexamethasone (1), etoposide (1), mycophenolic acid (1) |
| Vascular disorders | 3 (1.0) | Pegaspargase (2), rituximab (1) | Cytarabine (1), dexamethasone (1), etoposide (1), hydrocortisone (1), mercaptopurine (1), methotrexate (1) |
| Eye disorders | 2 (0.7) | Defibrotide (2) | Heparin (2) |
| Reproductive system and breast disorders | 1 (0.3) | *Erwinia* asparaginase (1) | Dexamethasone (1), methotrexate (1), vincristine (1) |
| Musculoskeletal and connective tissue disorders | 1 (0.3) | Imatinib (1) |  |
| Psychiatric disorders | 1 (0.3) | Eltrombopag (1) |  |

*More than one drug could be involved in an ADR.

**Table 6. Principal ADR identified during the follow-up with no drugs of the study list involved as suspicious***

| **ADR according to the affected system** | **n (%)** | **Suspicious drugs in ≥2 ADR** |
| --- | --- | --- |
| Pancytopenia | 20 (6.8) | Cytarabine (15), mercaptopurine (13), cyclophosphamide (7), dexamethasone (7), methotrexate (5), vincristine (4), doxorubicin (2) |
| Anemia | 18 (6.1) | Cytarabine (12), mercaptopurine (12), vincristine (5), cyclophosphamide (4), dexamethasone (4), doxorubicin (4) |
| Thrombocytopenia | 15 (5.1) | Cytarabine (10), mercaptopurine (10), cyclophosphamide (3), dexamethasone (2), doxorubicin (2), vincristine (2) |
| Stomatitis | 12 (4.1) | Dexamethasone (6), methotrexate (6), mercaptopurine (5), vindesine (5), doxorubicin (3), busulfan (2), cytarabine (2), fludarabine (2) |
| Hepatitis | 10 (3.4) | Cytarabine (6), methotrexate (6), dexamethasone (5), cyclophosphamide (4), mercaptopurine (3), mesna (3), vincristine (3) |
| Rhinovirus infection | 10 (3.4) | Dexamethasone (5), cytarabine (4), cyclosporine (3), methotrexate (3) |
| Vomiting | 10 (3.4) | Cyclophosphamide (5), mesna (4), mercaptopurine (2), methotrexate (2), pentamidine (2) |
| Febrile neutropenia | 9 (3.0) | Mercaptopurine (5), dexamethasone (3), doxorubicin (3), methotrexate (3), vincristine (3), cytarabine (2) |
| Acute renal failure | 8 (2.7) | Methotrexate (3), acyclovir (2), amlodipine (2), spironolactone (2), furosemide (2) |
| Agranulocytosis | 7 (2.4) | Cyclophosphamide (3), mercaptopurine (3), cytarabine (2), fludarabine (2) |
| Fever | 7 (2.4) | Mercaptopurine (6), cytarabine (5), methotrexate (3), cyclophosphamide (2), anti-thymocyte globulin (2) |
| Respiratory tract viral infection | 7 (2.4) | Mercaptopurine (5), methotrexate (4), cytarabine (2) |
| Other | 162 (54.8) |  |

*More than one drug could be involved in an ADR.
